# Effects of pharmacological and environmental manipulations on choice between fentanyl and shock avoidance/escape in male and female rats under mutually exclusive and non-exclusive choice conditions

**DOI:** 10.1038/s41386-024-01939-7

**Published:** 2024-08-05

**Authors:** Madison M. Marcus, Samuel A. Marsh, Michelle Arriaga, S. Stevens Negus, Matthew L. Banks

**Affiliations:** Department of Pharmacology and Toxicology, Virginia Commonwealth University School of Medicine, Richmond, VA USA

**Keywords:** Reward, Motivation, Preclinical research

## Abstract

Substance use disorders are defined by persistent drug consumption despite adverse consequences. Accordingly, we developed two fentanyl-vs-shock avoidance/escape choice procedures in which male and female rats responded under a fixed-ratio (FR)1:FR1 concurrent schedule of shock avoidance/escape and IV fentanyl under either mutually exclusive or non-exclusive choice conditions. Initial experiments using a discrete-trial procedure determined behavioral allocation between mutually exclusive shock avoidance/escape and different fentanyl doses (0.32–18 μg/kg/infusion; Experiment 1). Shock intensity (0.1–0.7 mA) and shock avoidance/escape response requirement (FR1-16) were also manipulated (Experiment 2). Next, we used a free-operant procedure in which shock avoidance/escape and fentanyl were continuously available under non-exclusive conditions, and response-shock (R-S) interval (30–1000 s) was manipulated (Experiment 3). Finally, we tested the hypothesis that extended-access fentanyl self-administration would produce fentanyl dependence, establish fentanyl withdrawal as an endogenous negative reinforcer, and increase fentanyl choice in both procedures (Experiments 4 and 5). The shock avoidance/escape contingency decreased fentanyl self-administration, and rats consistently chose shock avoidance/escape over fentanyl in both choice conditions. Decreasing shock intensity or increasing shock avoidance/escape response requirement failed to increase fentanyl choice, suggesting that fentanyl and shock avoidance/escape are independent economic commodities. Increasing the R-S interval increased fentanyl choice but failed to increase shock delivery. Extended fentanyl access engendered high fentanyl intake and opioid withdrawal signs but failed to increase fentanyl choice under either choice condition. These results suggest that neither positive fentanyl reinforcement nor negative reinforcement by fentanyl withdrawal is sufficient to reduce shock avoidance/escape-maintained responding and increase foot shock as an adverse consequence.

## Introduction

Continued addictive drug use despite adverse consequences is the hallmark behavioral characteristic of DSM-5 criteria for substance use disorders. Drug use despite adverse consequences in non-humans has been routinely studied using procedures in which operant responding results in a drug infusion paired with a putative aversive stimulus, such as electric foot shock [1]. Studies in both rodents and non-human primates consistently show that electric foot shock functions as a positive punisher to decrease cocaine [2–5] and methamphetamine [6] self-administration under a broad range of experimental conditions. However, in contrast to the rich literature on punished psychostimulant self-administration, fewer studies have examined punished opioid self-administration [7–10]. In addition, many of these published studies used single-operant self-administration procedures where the primary dependent measure is rate of responding and only a single reinforcer is available. The use of single-operant self-administration procedures may result in interpretive complications, some of which can be mitigated by preclinical drug-choice procedures (see ref. [11]).

In a typical drug-choice procedure, the subject has concurrent access to a drug and non-drug positive reinforcer (e.g., food or social interaction) [8, 12–14]. In this choice context, punished drug-taking behavior not only decreases drug-self administration but also promotes behavioral reallocation away from the punished reinforcer and towards the unpunished reinforcer [15–18]. Preclinical choice procedures have shown translational utility and predictive validity in part because they model a common human situation where more than one reinforcement option exists [11]. In addition to non-drug positive reinforcers, non-drug negative reinforcers are also available in our natural environment and may compete with behavior maintained by addictive drugs. A negative reinforcer is defined as a stimulus whose *removal* increases the likelihood of the response that preceded it [19]. In humans, non-drug alternative negative reinforcers may be available under either mutually exclusive choice conditions (i.e., an individual can use rent money to either purchase fentanyl or avoid eviction as a negative reinforcer) or under non-exclusive choice conditions (i.e., an individual may consume drug and still perform at work, avoiding termination and unemployment as a negative reinforcer).

Studies in rodents and nonhuman primates have demonstrated that scheduled electric shock functions as a negative reinforcer [20–22]. Accordingly, one goal of the present study was to investigate determinants of behavioral allocation between fentanyl and shock avoidance/escape in male and female rats under two fentanyl-vs-shock avoidance/escape choice procedures. In the mutually exclusive (i.e., dependent schedules), discrete-trial concurrent choice procedure, rats could respond during each trial either to avoid or escape a scheduled shock (i.e., choose negative reinforcement) or to receive an IV fentanyl infusion followed by the scheduled shock (i.e., choose the drug despite adverse consequence). In the non-exclusive (i.e., independent schedules), free-operant choice procedure, rats could respond to avoid/escape a scheduled shock and simultaneously respond for IV fentanyl infusions. Responding for shock avoidance/escape did not influence the availability of the fentanyl reinforcer and vice versa. We hypothesized that fentanyl self-administration would be lower in the mutually exclusive fentanyl-vs-shock avoidance/escape procedure than in the non-exclusive choice procedure. Subsequent parametric studies manipulated fentanyl dose, shock intensity, response requirement, and response-shock interval. In accordance with existing fentanyl-vs-positive reinforcer choice literature [23, 24], we hypothesized that increasing the fentanyl dose, decreasing the shock intensity, increasing the shock avoidance/escape response requirement, and increasing the response-shock (R-S) interval would promote behavioral allocation towards fentanyl and away from shock avoidance/escape.

A second goal of the study was to determine the effects of opioid dependence and withdrawal on fentanyl-vs-shock avoidance/escape choice. One contemporary addiction theory posits that opioid withdrawal functions as an endogenous negative reinforcer that can be avoided or terminated by further drug self-administration [25, 26]. Accordingly, we hypothesized that opioid withdrawal in opioid-dependent subjects would function as an endogenous negative reinforcer that would compete with shock as an exogenous negative reinforcer and thereby increase fentanyl choice in both the mutually exclusive and non-exclusive choice procedures. We further hypothesized that there would be a more pronounced increase in fentanyl choice under non-exclusive choice conditions compared to mutually exclusive choice conditions during opioid withdrawal.

## Methods

### Subjects

A total of 28 Sprague-Dawley rats (14 M, 14 F) weighing 240-310 g upon arrival were used. Fourteen rats (7 M, 7 F) were initially trained on negative reinforcement (i.e., foot shock avoidance/escape), ten rats (5 M, 5 F) were initially trained on fentanyl self-administration, and four rats (2 M, 2 F) were initially trained on food maintained responding as described previously [27]. A detailed description of operant training procedures is reported in Supplemental Materials. The training order of shock avoidance/escape, fentanyl self-administration, and food-maintained responding was counterbalanced between rats to address the potential influence of operant training history on behavior. Separate cohorts of rats were used for the mutually exclusive and non-exclusive choice studies. Final sample sizes are reported for each experiment. Animals were singly housed in a temperature and humidity-controlled vivarium and maintained on a 12-h light/dark cycle (lights off at 6:00 PM). Water and food (Teklad Rat Diet, Envigo) were provided ad-libitum in the home cage. Daily behavioral sessions were conducted Monday-Friday from approximately 7 AM–12 PM. Overnight sessions were conducted Sunday–Thursday from approximately 6 PM–6 AM. Animal maintenance and research were conducted in accordance with the 2011 Guidelines of the National Institutes of Health Committee on Laboratory Animal Resources. Both enrichment and research protocols were approved by the Virginia Commonwealth University Institutional Animal Care and Use Committee.

### Mutually exclusive fentanyl-vs-shock avoidance/escape choice procedure

Following successful training of both shock avoidance/escape and fentanyl-maintained responding alone, a cohort of rats (*n* = 20) was trained in the mutually exclusive (i.e., dependent schedules), discrete-trial fentanyl-vs-shock avoidance/escape choice procedure consistent with our previous publication [27]. Daily behavioral sessions consisted of two single-operant trials followed by nine discrete choice trials. Illumination and extinguishment of a tricolor light above the right, fentanyl-associated lever and a white light above the left, shock avoidance/escape-associated lever coincided with lever extension and retraction, respectively. Every response-requirement completion resulted in immediate retraction of the associated lever. A 4 min 27 s time-out period during which all levers were retracted separated each trial. The first trial was a fentanyl-only trial where response requirement (fixed-ratio: FR1) completion on the right, fentanyl-associated lever resulted in an IV infusion of the fentanyl dose available during the subsequent choice trials. No shock was administered during the fentanyl-only trial. The fentanyl only trial incorporated a 30-min limited hold meaning if the response requirement was not met within 30-min, a non-contingent IV infusion of the available fentanyl dose was administered. Following a time-out period, a shock avoidance/escape only trial was initiated, during which a 3-s shock stimulus (0.7 mA) was scheduled for presentation 30 s after trial onset. Response requirement (FR1) completion on the left, shock avoidance/escape-associated lever during the initial 30 s resulted in canceled shock presentation at the end of the trial (avoidance response), whereas response-requirement completion during the 3-s shock escaped the remainder of the shock (escape response). Following completion of both single-operant trials, a time-out period occurred, and choice trials were initiated. During each of the nine choice trials, both the fentanyl- and shock avoidance/escape-associated levers were extended for up to 33 s. During the initial 30-s, the rat could complete the response requirement on the fentanyl-associated lever for a fentanyl infusion immediately followed by a 3-s inescapable foot shock (0.7 mA) or the shock avoidance/escape-associated lever to cancel the upcoming shock stimulus (i.e., avoidance response). If the response requirement was not met on either lever during the 30-s avoidance period, a 3-s shock (0.5 mA) was presented. During the 3-s shock, response-requirement completion on the fentanyl-associated lever resulted in a fentanyl infusion without shock termination (e.g., full 3 s shock), whereas response requirement completion on the shock avoidance/escape-associated lever resulted in immediate shock termination (i.e., escape response, < 3 s shock). If the response requirement was not completed on either lever after 3 s of shock, all stimuli including shock were terminated, levers retracted, and the trial was recorded as an omission. Response-requirement (FR1) completion on the fentanyl-associated lever was counted as a fentanyl+shock trial and response-requirement completion (FR1) on the shock avoidance/escape-associated lever was counted as a shock avoidance/escape trial.

### Non-exclusive fentanyl-vs-shock avoidance/escape procedure

Following successful training of both shock avoidance/escape and fentanyl-maintained responding alone, a second cohort of rats (*n* = 8) was trained in the non-exclusive (i.e., independent schedules), free-operant fentanyl-vs-shock avoidance/escape choice procedure. In this one h procedure, both the fentanyl- and shock avoidance/escape-associated levers were extended, associated discriminative stimulus lights were illuminated, and a 0.5-s foot shock was scheduled to occur every 5-s (i.e., shock-shock interval of 5-s). Consistent with established free-operant avoidance/escape procedures [20], the rat could lever-press on the shock avoidance/escape-associated lever (FR1) to prolong the time until the next shock presentation according to the experimenter-determined response-shock (R-S) interval (30–1000 s). Response requirement completion on the avoidance/escape-associated lever did not result in lever retraction or extinction of the associated stimulus light. Concurrently, rats could respond on the fentanyl-associated lever for an IV infusion of the available fentanyl dose (0.32–3.2 µg/kg/infusion) under an FR1 / 20-s timeout schedule of reinforcement. During the timeout period, the fentanyl-associated lever was retracted, and the stimulus light was extinguished. The timeout was incorporated to mitigate subjects’ risk of opioid overdose. In contrast to the mutually exclusive choice procedure, responding for the fentanyl reinforcer did not influence negative reinforcer (shock avoidance/escape) availability and vice versa. Thus, the two schedules of reinforcement operated independently of each other and the only limit to the number of fentanyl or shock avoidance/escape responses was the session duration.

### Experiment 1: Effects of reinforcement schedule on fentanyl-vs-shock avoidance/escape choice

Experiment 1 determined the effects of concurrently available shock avoidance/escape on fentanyl self-administration under both mutually exclusive and non-exclusive choice conditions. Three fentanyl doses were tested in each fentanyl-vs-shock avoidance/escape choice procedure to determine a dose-effect function. Fentanyl dose in the mutually-exclusive choice procedure (3.2, 10 and 18 µg/kg/infusion) and non-exclusive choice procedure (0.32, 1 and 3.2 µg/kg/infusion) was varied by changing the infusion duration (e.g., 300 g rat: 5, 15, and 27-s syringe pump activation, respectively) and fentanyl dose order was counterbalanced between rats. In the mutually-exclusive choice procedure, each fentanyl dose was evaluated for two days with the shock avoidance/escape intensity at 0.7 mA. The results from both test days were averaged and used for data analysis. In the non-exclusive choice procedure, each fentanyl dose was evaluated for three days and the results from the final two test days were averaged and used for data analysis. After completing the dose-response curve at a 30 s R-S interval, the dose-response curve was redetermined using the same methods at the 100 s R-S interval in the non-exclusive choice procedure. In the non-exclusive choice procedure, shock intensity for each rat was individualized and equivalent to the final shock intensity used at the end of single operant shock avoidance/escape training (mean: 0.5 mA [range: 0.4–0.6 mA]).

Following the completion of dose-effect functions under both choice procedures, we determined whether the removal of the concurrent shock avoidance/escape contingency would increase fentanyl self-administration. In the mutually exclusive choice procedure, fentanyl (3.2 and 10 µg/kg/infusion) -vs-shock avoidance/escape (0.5 mA) choice was re-determined for five consecutive days. Then, the shock avoidance/escape contingency and associated discriminative stimuli (light and lever extension) were removed from the choice procedure resulting in a nine discrete-trial fentanyl self-administration procedure. Under these conditions, only the fentanyl-associated lever and stimulus light were presented and there were no scheduled shocks, similar to the initial fentanyl-only trial. The number of trials completed for 3.2 and 10 µg/kg/infusion fentanyl were determined for five consecutive days and dose order was counterbalanced between rats. The 10 µg/kg/infusion fentanyl dose was utilized for all subsequent mutually exclusive choice experiments. In the non-exclusive choice procedure, choice between 0.32 µg/kg/infusion fentanyl and no scheduled shock was determined for three consecutive days. Under these conditions, the shock avoidance/escape-associated lever was still extended, and discriminative stimulus light was still turned on, but no shocks were scheduled. The 0.32 µg/kg/infusion fentanyl dose was utilized for all subsequent non-exclusive choice experiments.

### Experiment 2: Effects of shock intensity and response requirement on mutually exclusive fentanyl-vs-shock avoidance/escape choice

Experiment 2 A determined the effects of different shock intensities on behavioral allocation between shock avoidance/escape and 10 µg/kg/infusion fentanyl in the mutually exclusive choice procedure. Shock intensity was decreased and then increased (0.7, 0.5, 0.3, 0.1, 0.3, 0.5, 0.7 mA) across seven consecutive test days. Response requirements (FR1) and fentanyl dose (10 µg/kg/infusion) was held constant. Experiment 2B determined the effects of increasing the shock avoidance/escape response requirement (FR 1, 2, 4, 8, 16) across five consecutive days. Shock intensity (0.5 mA), fentanyl dose (10 µg/kg/infusion), and fentanyl response requirement (FR1) were held constant. Based on Experiment 2A results, the 0.5 mA shock intensity was used in subsequent mutually exclusive fentanyl-vs-shock avoidance/escape choice experiments.

### Experiment 3: Effects of R-S interval on non-exclusive fentanyl-vs-shock avoidance/escape choice

Experiment 3 determined effects of manipulating R-S interval on behavioral allocation between shock avoidance/escape and 0.32 µg/kg/infusion fentanyl in the non-exclusive choice procedure. Each R-S interval (30, 100, 300, 1000 s) was tested for three consecutive days and the average of the final two test days are shown. R-S intervals were tested in ascending order. In the “No shock” condition, responding on the avoidance/escape-associated lever had no programmed consequences and no shocks were scheduled.

### Experiment 4: Effects of opioid dependence and withdrawal on mutually exclusive fentanyl-vs-shock avoidance/escape choice

Experiment 4 determined the effects of daily extended-access fentanyl self-administration and subsequent opioid withdrawal on mutually exclusive fentanyl-vs-shock avoidance/escape choice and an experimental timeline is shown in Fig. 4A. Baseline fentanyl-vs-shock avoidance/escape choice was determined for five consecutive days and rats were weighed and scored for the presence of nine somatic withdrawal signs (tremor, teeth chatter, eye twitch, mastication, yawn, wet dog shake, piloerection, diarrhea, ptosis) [28, 29]. Subsequently, 12 h extended-access fentanyl self-administration sessions were introduced Sunday–Thursday for two consecutive weeks in addition to daily fentanyl-vs-shock avoidance/escape choice sessions conducted Monday–Friday. The extended-access fentanyl session occurred from 6PM – 6AM and rats could respond for an infusion of 3.2 µg/kg/infusion fentanyl under a FR1 / 10-s time-out schedule of reinforcement. Fentanyl-vs-shock avoidance/escape choice sessions were conducted at approximately 10:30 AM. Body weights and opioid somatic withdrawal signs were assessed daily immediately prior to the choice session. Following two weeks of extended fentanyl access, daily fentanyl-vs-shock avoidance/escape choice sessions continued for one additional week to track choice behavior following extended fentanyl access. Data collected on the Friday after termination of extended access served as the post-extended access data point for analyses.

### Experiment 5: Effects of opioid dependence and withdrawal on non-exclusive fentanyl-vs-shock avoidance/escape choice

Experiment 5 determined the effects of daily extended-access fentanyl self-administration and subsequent opioid withdrawal on non-exclusive fentanyl-vs-shock avoidance/escape choice. The order of the five R-S intervals (30 s, 100 s, 300 s, 1000 s, and no shock) was randomized and counterbalanced between rats according to a Latin square design. Thus, during each 5-day choice test period (baseline, extended access week 1, extended access week 2, post-extended access), each individual subject was tested in all five R-S interval conditions. Fentanyl-vs-shock avoidance/escape choice sessions were conducted at approximately 11:00 AM Monday–Friday of each week and body weights and opioid somatic withdrawal signs were assessed daily immediately prior to the choice session. Following the determination of baseline fentanyl-vs-shock avoidance/escape choice, the extended-access fentanyl sessions (6PM–6AM Sunday–Thursday) were introduced in addition to the daily choice sessions. During the extended access session, rats could respond for an infusion of 3.2 µg/kg/infusion fentanyl under a FR1 / 10-s time-out schedule of reinforcement. Following two weeks of extended fentanyl access, extended access sessions were discontinued, and daily fentanyl-vs-shock avoidance/escape choice sessions continued for one additional week to serve as the post-extended-access timepoint.

### Data analysis

The primary dependent measures in the mutually exclusive fentanyl-vs-negative reinforcer choice procedure were: (1) fentanyl+shock trials completed, (2) shock avoidance/escape trials completed, and (3) omitted trials. The primary dependent measures in the non-exclusive fentanyl-vs-negative reinforcer choice procedure were: (1) fentanyl responses, (2) shock avoidance/escape responses, (3) shocks received, and (4) percent fentanyl choice defined as (number of fentanyl responses/total number of responses completed on both levers) × 100. These measures were plotted as a function of unit fentanyl dose or independent variable manipulation. Other dependent measures included the latency to complete the first fentanyl-only forced trial in the mutually exclusive choice procedure, the number of fentanyl infusions earned during extended-access sessions, and opioid somatic withdrawal signs. Data were analyzed using repeated-measures one- or two-way analysis of variance, mixed-effect analysis, paired *t*-test, or Friedman test, as appropriate. For mutually exclusive choice data analysis, the main factors were reinforcer (fentanyl or shock avoidance/escape) trials and independent variable manipulation. Omitted trials were analyzed separately. Sphericity violations were corrected using the Greenhouse-Geisser epsilon. Significant main effects or interactions were followed by planned post-hoc tests that corrected for multiple comparisons. The criterion for significance was set a priori at the 95% level of confidence (*p* < 0.05), and all analyses were conducted using GraphPad Prism (v 10.0.0, La Jolla, CA).

### Drugs

Fentanyl HCl was supplied by the NIDA Drug Supply Program (Bethesda, MD). Fentanyl was dissolved in sterile saline for injection and passed through a 0.22-micron sterile filter before IV administration. Fentanyl doses are expressed as the salt form listed above.

## Results

### Experiment 1: Effects of reinforcement schedule on fentanyl-vs-shock avoidance/escape choice

**Fig. 1:**
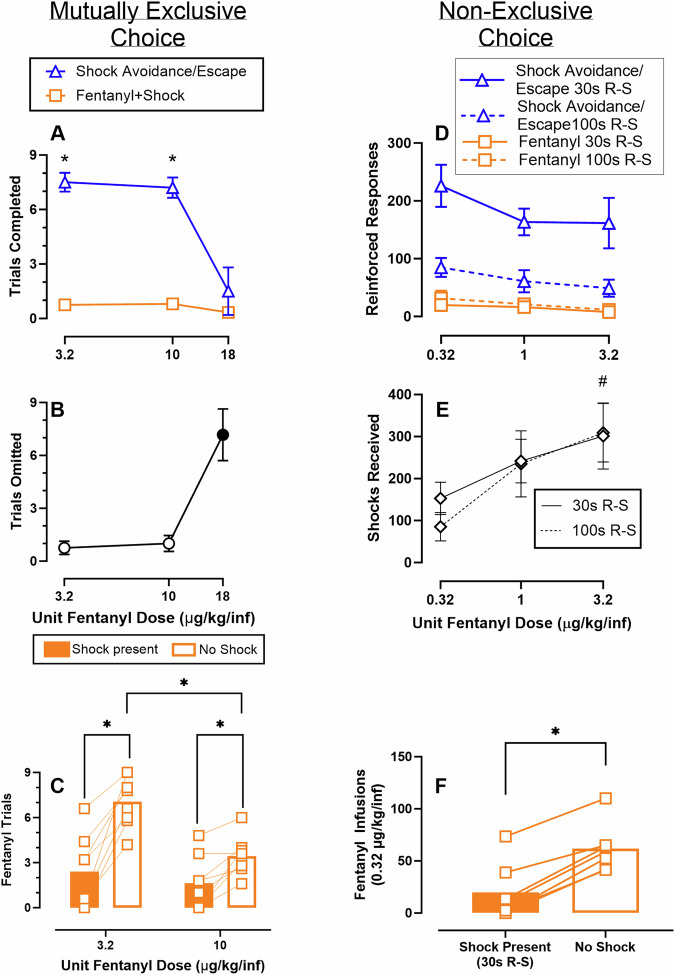
Behavioral allocation between fentanyl and the negative reinforcer of shock avoidance/escape under mutually exclusive and non-exclusive concurrent choice conditions as a function of unit fentanyl dose and reinforcer availability. **A** Trials completed for fentanyl+shock (FR1) or shock avoidance/escape (FR1, 0.7 mA) in a nine discrete-trial, mutually exclusive fentanyl-vs-shock avoidance/escape procedure as a function of fentanyl dose. * denotes significant (*p* < 0.05) difference between fentanyl+shock and shock avoidance/escape at a given fentanyl dose. **B** Trials omitted in the mutually exclusive fentanyl-vs-shock avoidance/escape procedure as a function of fentanyl dose. Filled symbol denotes significant difference compared to 3.2 µg/kg/inf fentanyl. **C** Fentanyl trials completed (FR1) as a function of fentanyl dose in the mutually exclusive choice procedure when shock avoidance/escape (0.5 mA) and associated discriminative stimuli were present (filled bars) or absent (open bars) * denotes significant difference. **D** Reinforced responses for fentanyl (FR1) or shock avoidance/escape (FR1, mean shock intensity 0.5 mA [0.4–0.6 mA]) in a non-exclusive fentanyl-vs-shock avoidance/escape choice procedure as a function of fentanyl dose, grouped by R-S interval. **E** Shocks received as a function of fentanyl dose in a non-exclusive fentanyl-vs-shock avoidance/escape procedure under two R-S conditions. ^#^ denotes significant difference from 0.32 µg/kg/inf fentanyl collapsed across R-S conditions. **F** Fentanyl infusions earned (0.32 µg/kg/inf, FR1) when the shock stimulus (mean 0.5 mA) is present (filled bar) or absent (open bar) * denotes significant difference. **A**, **B**, **D**, **E** Points and bars represent group mean ± SEM. **C**, **F** Points represent individual subjects, bars represent group mean. **A** *n* = 6–10 (2–4 M/4-6 F); **B**, **C** *n* = 8 (4 M/4 F); **D**–**F** *n* = 7 (4 M/3 F).

27 out of 28 rats successfully acquired the shock escape response. In the discrete-trial avoidance training, only one female rat successfully acquired the shock avoidance response. The avoidance contingency was continued throughout the mutually exclusive choice experiments to determine if any manipulations altered avoidance responding. Overall, avoidance responding was extremely low, accounting for < 5% of the negative reinforcement trials completed throughout all experiments. Figure 1A, B shows avoidance/escape and fentanyl+shock trials completed, or trials omitted, across a range of fentanyl doses (3.2–18 µg/kg/infusion) in the mutually exclusive choice procedure. Significantly more shock avoidance/escape than fentanyl+shock trials were completed at both 3.2 and 10 µg/kg/infusion doses (dose: F(1, 9.2) = 23.8, *p* = 0.0008; reinforcer type: F(1, 9) = 75.7, *p* < 0.0001); dose × reinforcer: F(1.7, 5.1) = 17.8, *p* = 0.0056). The number of fentanyl+shock trials completed across all fentanyl doses tested was approximately one trial. Increasing unit fentanyl doses resulted in increased omitted trials, with 18 µg/kg/infusion fentanyl significantly increasing omitted trials compared to 3.2 µg/kg/infusion fentanyl (dose: F(1, 6.1) = 23.5, *p* = 0.0026). Females completed significantly fewer shock avoidance/escape trials and omitted more trials compared to males in the mutually exclusive choice procedure (Fig. S1). Figure 1C shows that removal of the shock avoidance/escape contingency and discriminative stimuli significantly increased the number of fentanyl trials completed for both 3.2 and 10 µg/kg/infusion fentanyl doses (dose: F(1,7) = 16, *p* = 0.0052; shock avoidance/escape presence or absence: F(1,7) = 55.1, *p* = 0.0001; dose × shock avoidance/escape: F(1,7) = 13, p = 0.0086). Collapsed across shock conditions, females completed more trials for 3.2 µg/kg/infusion fentanyl compared to males (Fig. S2). Additionally, removal of the shock avoidance/escape contingency decreased the latency to complete the first fentanyl-only trial regardless of fentanyl dose (Fig. S2). Pretreatment with diazepam (0.32–5.6 mg/kg, IP) failed to alter mutually exclusive fentanyl-vs-shock avoidance/escape choice up to doses that increased omissions (see Supplementary Materials and Figs. S3–4). Figure 1D shows reinforced responses for both shock avoidance/escape and fentanyl across a range of fentanyl doses (0.32–3.2 µg/kg/infusion) and two response-shock (R-S) conditions (30, 100 s) in the non-exclusive choice procedure. Compared to the 30 s R-S condition, fewer responses were made on the shock avoidance/escape lever (R-S interval: F(1, 6) = 21.0, *p* = 0.0038) and more responses were made on the fentanyl-associated lever (R-S interval F(1, 6) = 10.6, *p* = 0.0172) in the 100 s R-S condition. There was no significant effect of dose on either the number of shock avoidance/escape or fentanyl responses emitted. Figure 1E shows shocks received across a range of fentanyl doses (0.32–3.2 µg/kg/infusion) and R-S conditions in the non-exclusive choice procedure. There were significantly more shocks received at the 3.2 µg/kg/infusion fentanyl dose compared to the 0.32 µg/kg/infusion fentanyl dose, collapsed across R-S conditions (fentanyl dose: F(1.8, 10.8) = 5.5, *p* = 0.0254). This is consistent with experimenter observations and event logs (Figs. S5–6) during 3.2 μg/kg/infusion availability where rats stopped responding altogether after self-administering enough fentanyl to produce motor impairment and continued to receive hundreds of shocks. Females took significantly more fentanyl infusions under 100 s R-S conditions and received significantly more shocks under 30 s R-S conditions compared to males (Fig. S7). Figure 1F shows that 0.32 µg/kg fentanyl infusions significantly increased in the non-exclusive choice procedure under 30 s R-S conditions upon removal of the shock avoidance/escape contingency (t(6) = 12, *p* < 0.001). There were no sex differences (Fig. S8).

### Experiment 2: Effects of shock intensity and response requirement manipulations on mutually exclusive fentanyl-vs-shock avoidance/escape choice

**Fig. 2:**
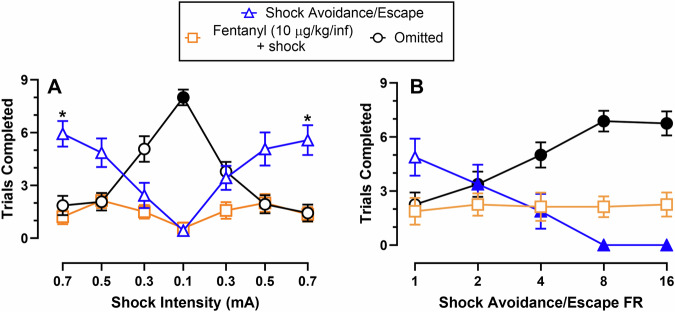
Effects of manipulating shock intensity and response requirement on fentanyl-vs-shock avoidance/escape choice in a mutually exclusive, discrete-trial choice procedure. **A** Trials completed for fentanyl+shock (FR1), shock avoidance/escape (FR1), or omissions as a function of shock intensity. * denotes a significant (*p* < 0.05) difference between fentanyl+shock and shock avoidance/escape within a given shock intensity. Filled symbols denote a significant difference in omissions at 0.1 mA compared to all other shock intensities. **B** Trials completed for fentanyl+shock, shock avoidance/escape (0.5 mA), or omissions as a function of shock avoidance/escape response requirement. Filled symbols denote significant difference within a reinforcer or omission compared to FR1. All points represent mean ± SEM; **A** *n* = 14 (7 M/7 F); **B** *n* = 8 (4 M/4 F).

Figure 2 shows effects of avoidance/escape shock intensity (2A) or response requirement (2B) manipulations on mutually exclusive fentanyl-vs-shock avoidance/escape choice. Figure 2A shows that 0.7 mA shock avoidance/escape was significantly chosen over 10 µg/kg/infusion fentanyl+shock (shock intensity: F(4.2, 54.1) = 18, *p* < 0.0001; reinforcer type: F(1, 13) = 10.1, *p* = 0.0072; shock intensity × reinforcer type: F(4.6, 60) = 5.2, *p* = 0.0007). As shock intensity decreased, omitted trials increased (F(3.6, 47) = 25.2, *p* < 0.0001), and significantly more trials were omitted at the 0.1 mA shock intensity compared to all other shock intensities. Figure 2B shows that increasing the shock avoidance/escape response requirement significantly decreased shock avoidance/escape trials completed without altering fentanyl+shock trials completed (response requirement: F(2,14.2) = 12.2, *p* = 0.0008; response requirement × reinforcer type: F(1.6, 11.3) = 6.9, *p* = 0.01). Omitted trials also increased with increasing shock avoidance/escape response requirement (F(2.2,15.6) = 23.6, *p* ≤ 0.0001). However, this increase in omitted trials was not due to an overall depression of behavior. Subjects continued responding on the shock avoidance/escape lever throughout the experiment but failed to satisfy the response requirement under the time constraints (Fig. S9). Females completed more fentanyl+shock trials and males completed more avoidance/escape trials across shock intensities (Fig. S10). No sex differences were detected across response requirement manipulations (Fig. S11).

### Experiment 3: Effects of R-S manipulations on non-exclusive fentanyl-vs-shock avoidance/escape choice

**Fig. 3:**
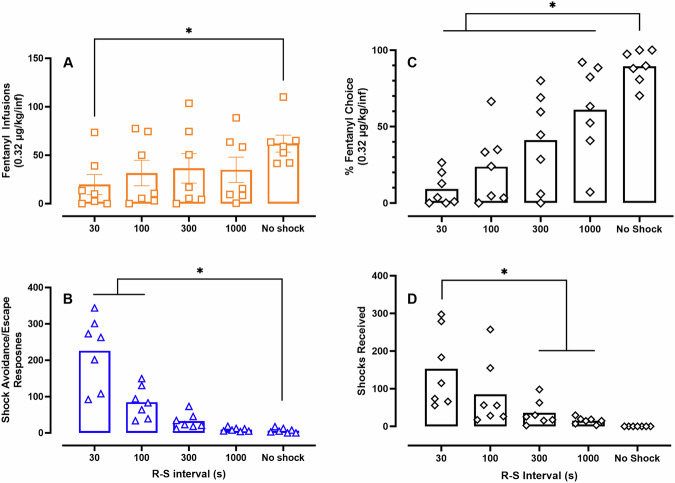
Effects of manipulating response-shock (R-S) interval on fentanyl-vs-shock avoidance/escape choice in a non-exclusive, free-operant choice procedure. Abscissae: R-S interval. **A** Number of 0.32 μg/kg/infusion fentanyl responses. **B** Number of shock avoidance/escape responses. **C** Percent fentanyl choice. **D** Shocks received. *denotes significant (*p* < 0.05) multiple comparisons. Points represent individual subjects (*n* = 7, 4 M/3 F) and bars represent group mean.

Figure 3A shows that 0.32 µg/kg fentanyl infusions increased with increasing R-S intervals (F(1.7, 10) = 10.6, *p* = 0.0044) and rats self-administered significantly more fentanyl under “No shock” conditions compared to the 30s R-S condition in the non-exclusive choice procedure. Inversely, responding on the shock avoidance/escape-associated lever decreased with increasing R-S interval (Fig. 1B; F(1.3, 7.8) = 33.0, *p* = 0.0003) and rats responded at higher rates under 30 and 100 s R-S conditions compared to the “No shock” condition. Complementing these changes in shock avoidance/escape responding, percent fentanyl choice was significantly higher under “No shock” conditions compared to all R-S intervals tested (Fig. 3C; F(2.2, 13.1) = 31.0, *p* < 0.0001). Fewer shocks were received under the 300 and 1000 s R-S interval compared to the 30 s R-S interval (F(1.5, 9.3) = 10.5, *p* = 0.0057; “No shock” condition excluded in analysis). Regarding the number of shocks received, there was a significant main effect of R-S interval, sex, and a R-S interval × sex interaction, but post-hoc multiple comparisons failed to detect significant differences (Fig. S12).

### Experiment 4: Effects of extended-access fentanyl on mutually exclusive fentanyl-vs-shock avoidance/escape choice

**Fig. 4:**
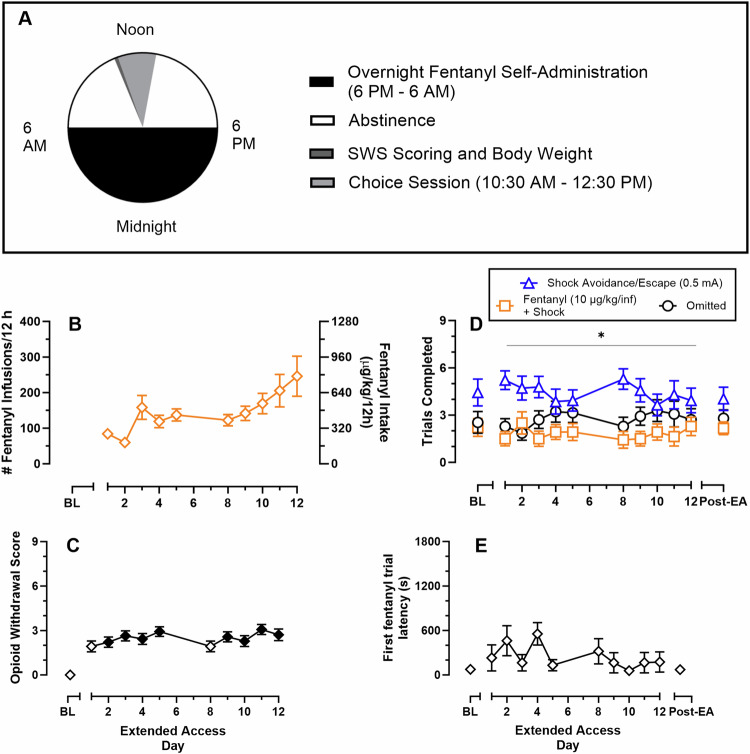
Effects of extended fentanyl access on somatic opioid withdrawal score, fentanyl-vs-shock avoidance/escape choice, and start latency in a mutually exclusive, discrete-trial choice procedure. Abscissae: experimental day. **A** Schematic of experimental design. **B** Number of fentanyl infusions earned (FR1 / TO10 s, 3.2 µg/kg/inf) and corresponding fentanyl intake during the extended-access self-administration session. **C** Opioid somatic withdrawal scores as a function of extended access day. **D** Trials completed for shock avoidance/escape, fentanyl+shock, or omissions during the mutually-exclusive fentanyl-vs-shock avoidance/escape choice procedure. **E** Latency in s to complete the initial fentanyl-only trial as a function of extended access day. Filled points denote significant (*p* < 0.05) difference compared to baseline. ^#^ indicates a significant difference between shock avoidance/escape and fentanyl regardless of extended access day. All points represent mean ± SEM, *n* = 14 (8 M/6 F). BL baseline, EA extended access.

Figure 4A shows the experimental timeline for the 12 h extended-access and mutually exclusive fentanyl-vs-shock avoidance/escape choice sessions. Rats self-administered more fentanyl over successive extended-access sessions (slope significantly different from zero: F(1,138) = 20.8, *p* < 0.0001; extended-access day: F(1.9, 25) = 6.5, *p* = 0.006); however, post-hoc analysis correcting for multiple comparisons failed to detect a significant difference between any extended-access day relative to Day 1. Maximal fentanyl intake during the extended-access session reached approximately 800μg/kg/12 h (Fig. 4B) and resulted in opioid dependence as demonstrated by a significant increase in somatic withdrawal signs (Fig. 4D; Friedman statistic: 51.1, *p* < 0.0001) and decreased bodyweight (Fig. S 13B; F(2.2, 27.6) = 22.2, *p* < 0.0001) compared to before the extended-access sessions. Figure 4C shows more shock avoidance/escape trials were completed compared to fentanyl+shock trials averaging across extended-access day (reinforcer type: F(1, 13) = 10.2, *p* = 0.007). Omitted trials remained consistent across days. Additionally, extended fentanyl access did not significantly alter the latency to complete the initial fentanyl-only trial (Fig. 4E). No sex differences were detected (Figs. S13–14).

### Experiment 5: Effects of extended-access fentanyl on non-exclusive fentanyl-vs-shock avoidance/escape choice

**Fig. 5:**
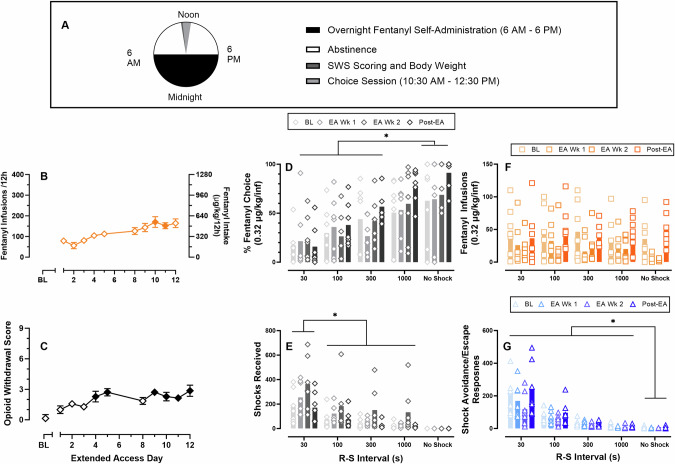
Effects of extended fentanyl access on somatic opioid withdrawal score and fentanyl-vs-shock avoidance/escape choice in a non-exclusive, free-operant choice procedure. Abscissae: **A**, **B** experimental day; **C**–**F** response-shock interval. **A** Schematic of experimental design. **B** Number of fentanyl infusions earned (FR1 / TO10 s, 3.2 µg/kg/inf) and corresponding fentanyl intake during the extended-access self-administration session. Filled symbols denote significant difference from Day 1. **C** Opioid somatic withdrawal scores. Filled symbols denote significant difference from BL. **D** Percent fentanyl choice. **E** Shocks received. **F** Number of fentanyl infusions earned during the choice session. **G** Number of shock avoidance/escape responses during the choice. Points represent individual subjects (*n* = 7, 4 M/3 F); bars represent group mean. Brackets represent significant multiple comparisons between R-S conditions. BL baseline, EA extended access.

Figure 5A shows the experimental timeline for the 12 h extended-access and non-exclusive fentanyl-vs-negative reinforcer choice sessions. Rats self-administered more fentanyl during extended access sessions on experimental days 10 and 11 compared to day 1 (day: F(2.8, 16.1) = 12.1, *p* = 0.0003) with maximal fentanyl intake reaching approximately 550 μg/kg/12 h (Fig. 5B). Extended access fentanyl resulted in opioid dependence as demonstrated by a significant increase in somatic withdrawal signs (Fig. 5C; Friedman statistic: 32.41, *p* = 0.003) and decreased bodyweight (Fig. S15B; F(1.3, 7.7) = 39.6, *p* = 0.0002) compared to before the extended-access sessions. There was no effect of extended access day on any endpoints shown in Fig. 5D–G (data not shown). Increasing the R-S interval increased percent fentanyl choice (Fig. 5D; F(2.1,12.5) = 16.8, *p* = 0.0003), decreased shocks received (Fig. 5E; F(1.6, 9.5) = 24.3, *p* = 0.0003), and decreased shock avoidance/escape responses (Fig. 5G; F(1.0, 6.1) = 21.8, *p* = 0.0033). There was no significant effect of extended access week on percent fentanyl choice, shocks received, or shock avoidance/escape responses (Fig. 5D, E, G). There was a significant effect of extended access week on fentanyl infusions earned during the choice session (Fig. 5F; F(1.4, 8.2) = 4.9, *p* = 0.049) but post-hoc tests failed to detect significant multiple comparisons. Relative to baseline conditions and post-extended access conditions, there was a trend towards increased shocks received and decreased shock avoidance/escape and fentanyl responses during extended access weeks. This trend was consistent with experimenter observations of decreased motor activity in the choice sessions during extended access weeks. Additionally, relative to baseline, there was a trend towards increased percent fentanyl choice and an increased number of fentanyl infusions post-extended access under 1000s R-S and “No Shock” conditions. Similar trends were observed in both males (Fig. S16) and females (Fig. S17).

## Discussion

The present study aim was to improve our fundamental knowledge in the behavioral processes involved in drug-taking despite adverse consequences [30] using both a mutually exclusive and non-exclusive fentanyl-vs-shock avoidance/escape choice procedure in female and male rats. There were three main findings. First, the concurrent availability of shock avoidance/escape as a negative reinforcer decreased fentanyl self-administration across a range of fentanyl doses under both mutually exclusive and non-exclusive choice conditions. In addition, decreasing the intensity or increasing the response requirement for shock avoidance/escape failed to promote behavioral reallocation away from shock avoidance/escape and towards fentanyl, and generally resulted in increased omitted trials. This suggests that shock avoidance/escape and IV fentanyl infusions might function as independent economic commodities rather than economic substitutes. Second, increasing the R-S interval in the non-exclusive choice procedure increased fentanyl choice. Thus, fentanyl-vs-negative reinforcer choice was sensitive to environmental manipulations under non-exclusive choice conditions. Finally, expression of opioid dependence and withdrawal failed to promote behavioral reallocation away from shock avoidance/escape and towards fentanyl under either mutually exclusive or non-exclusive choice conditions. Thus, even under conditions of opioid withdrawal proposed by contemporary addiction theories to function as a negative reinforcer of fentanyl self-administration, the strength of this *hypothesized* endogenous negative reinforcement (i.e., escape from fentanyl withdrawal) was still weaker than electric shock as an exogenous negative reinforcer, and fentanyl self-administration still failed as an economic substitute for shock avoidance/escape. Overall, the present results demonstrate that concurrent negative reinforcement contingencies decrease opioid self-administration under some experimental conditions. However, the therapeutic utility of negative reinforcement to compete with opioid self-administration in humans may be limited.

### Behavioral economic considerations of fentanyl and a negative reinforcer as commodities

Previous opioid choice studies in both nonhuman primates and rats with concurrently available positive reinforcers such as liquid food [24], food pellets [31], or social interaction [23, 32] have demonstrated that opioid choice was sensitive to reinforcer magnitude, such that opioid choice increased with either increasing opioid doses or decreasing magnitudes of the nondrug alternative. Similar results have also been reported in human laboratory opioid choice studies where the alternative nondrug positive reinforcer was money [33–35]. One interpretation from this literature is that opioid reinforcers and these nondrug positive reinforcers are economic substitutes [35–37]; as the magnitude of one reinforcer increases, consumption of the other reinforcer decreases proportionally. However, this pattern of behavioral allocation was absent in the current fentanyl-vs-shock avoidance/escape studies. Under the mutually exclusive choice condition, no fentanyl dose was chosen over shock avoidance/escape, even when fentanyl was available at relatively high doses (>0.32 µg/kg/inf) sufficient to maintain choice of fentanyl over food and social interaction as positive reinforcers [23, 24]. Similarly, neither decreases in shock intensity (i.e., negative reinforcer magnitude) nor increases in shock avoidance/escape response requirement facilitated behavioral reallocation away from shock avoidance/escape and towards fentanyl choice and instead resulted in increased omitted trials. Even at high avoidance/escape response requirements, rats continued to respond on the shock avoidance/escape lever (Fig. S9), suggesting that omitted trials were due to ratio strain, not behavioral depression. These results suggest that fentanyl and shock avoidance/escape are economic independents rather than substitutes [36, 37] such that rats either allocated behavior towards shock avoidance/escape or omitted trials.

### Implications for preclinical models of drug-taking despite adverse consequences

Most preclinical studies modeling drug-taking despite adverse consequences have incorporated stimuli as positive punishers of drug-taking behavior in the absence of alternative nondrug reinforcers [1, 30, 38]. In these studies, shock was scheduled as a consequence of the same behavior that produced the positive reinforcer and could only be avoided by withholding responses for the positive reinforcer [10, 39]. In the present study, shock was scheduled not as a punisher of fentanyl self-administration but as an alternative negative reinforcer, such that withholding responses on the fentanyl-associated lever was insufficient to avoid shock. Instead, shock could be avoided or escaped only by actively emitting behavior on the negative reinforcement-associated lever. Under these conditions, there was little evidence of drug-taking despite adverse consequences, and rats allocated almost all their behavior towards shock avoidance/escape. A hint of fentanyl-taking despite adverse consequences was only observed at the largest fentanyl dose tested (3.2 μg/kg/infusion) in the free-operant choice procedure. At this fentanyl dose, shock presentations increased and fentanyl and shock avoidance/escape-maintained responding remained stable (Fig. 1). Examination of the session event logs (Figs. S5–6) reveals a more complex picture. Rats self-administered sufficient amounts of 3.2 μg/kg/infusion fentanyl early in the session to produce grossly observable motor impairment and long intervals during which they emitted no operant responses for either reinforcer, leading to the delivery of hundreds of shocks until session termination. Notably, this increase in shock delivery did not occur because rats were actively engaged in fentanyl self-administration at the expense of responding for shock avoidance/escape. Rather, it occurred under conditions of fentanyl-induced motor impairment that suppressed almost all responding (Figs. S5–6).

This finding agrees with a previous study that also examined choice between an opioid and avoidance/escape from scheduled shock (25-sec R-S) under non-exclusive choice conditions [40]. In that study, FR manipulations that increased heroin self-administration also increased shock presentations as responding for shock avoidance or escape decreased. This increase in heroin choice and reduction in shock avoidance/escape choice was interpreted as evidence of economic substitutability between these two reinforcers. However, both the heroin unit dose in that study (20 or 30 µg/kg/infusion) and the fentanyl unit dose that increased shock presentations in the present study (3.2 µg/kg/infusion) are equipotent to produce reinforcer-independent rate-altering effects that impair operant responding [24, 41, 42]. Taken together, these findings suggest that fentanyl and other addictive drugs may increase adverse consequences not because they are economic substitutes for negative reinforcers, but rather because they are economically independent commodities that remain reinforcing even at high doses that produce reinforcement-independent rate-altering effects and sustained impairment of all behavior, including behavior maintained by negative reinforcers.

### Implications for the role of negative reinforcement in addiction research

One contemporary addiction research theory proposes that, with continued addictive drug consumption, behavior initially maintained by the positive reinforcing effects of drug consumption continues not because of the positive reinforcing effects of the drug, but because continued drug consumption alleviates a hypothesized negative internal state [25, 26]. According to this theory, the fentanyl-vs-shock avoidance/escape procedure in fentanyl-dependent rats undergoing withdrawal could be conceptualized as choice between two negative reinforcers: (1) opioid withdrawal as an endogenous negative reinforcer that could be escaped by responding on the fentanyl lever, or (2) shock as an exogenous negative reinforcer that could be avoided/escaped by responding on the alternative lever. Although the present results do not exclude a role for opioid withdrawal as a negative reinforcer modulating fentanyl self-administration, these findings do suggest that fentanyl withdrawal is still a weaker negative reinforcer than shock avoidance/escape under both mutually exclusive and non-exclusive choice conditions and that withdrawal avoidance/escape via fentanyl self-administration fails to substitute for shock avoidance/escape. Further empirical experimentation testing of this theory [26] would enhance our fundamental understanding of drug reinforcement processes during periods of opioid withdrawal.

### Translational implications for the role of negative reinforcement in treatment strategies

Concurrently available positive reinforcers (e.g., food, social interaction) decrease opioid self-administration under both mutually exclusive [24, 31, 43] and non-mutually exclusive choice conditions [44, 45]. These findings from preclinical [24, 31, 43–45] and human choice studies [33–35] have informed the development of effective behavioral therapies for substance use disorder. Contingency management in which a non-drug positive reinforcer (e.g., money) is offered as an alternative to drug is one example of a behavioral treatment [46, 47]. The present results demonstrate that concurrently available negative reinforcement (shock avoidance/escape) is also effective in decreasing fentanyl self-administration across many conditions including a broad range of fentanyl doses, high shock avoidance/escape response requirements, small shock magnitudes, and opioid withdrawal. This result is also consistent with recent cocaine-vs-shock avoidance/escape results [27]. Taken together, these results might suggest that, like positive reinforcers, scheduling negative reinforcers as an alternative to drug-taking behavior within a clinical population may reduce or compete with human addictive drug-taking behavior. However, there are several important caveats to this conclusion which limit the translational utility of using negative reinforcers as alternatives to addictive drugs. Three caveats will be discussed.

First, results from Experiment 3 showed that as the R-S interval increased, behavioral allocation towards a small fentanyl dose (0.32 µg/kg/infusion) increased. In fact, subjects earned the same number of fentanyl infusions under R-S intervals greater than 30 s as they did under no shock conditions. Thus, small fentanyl doses maintained high levels of behavioral allocation when the negative reinforcer was presented infrequently. This is a key finding because long R-S intervals might be most analogous to negative reinforcement contingencies in the clinical situation where a negative reinforcer (e.g., eviction due to failure to pay monthly rent) is encountered infrequently.

Second, only shock was used as the negative reinforcer in the present study, and it remains to be determined whether other potential negative reinforcers (e.g., histamine infusion) would also decrease opioid-taking behavior in a preclinical choice procedure. Shock would not be utilized as a negative reinforcer in humans; however, scheduled withdrawals from an escrow account would be one example of a negative reinforcer for humans. Although there are potential practical and ethical issues with this approach, these scheduled deductions could be postponed upon the patient engaging in non-drug related behaviors such as attending cognitive behavioral therapy sessions. Thus, despite the robustness of a concurrently available negative reinforcement contingency (shock avoidance/escape) to decrease fentanyl self-administration, the translational applications of using negative reinforcement to diminish addictive opioid-taking behavior appear limited.

Lastly, the present study was conducted in a simplified environment with only the negative reinforcer (shock avoidance/escape) and IV fentanyl concurrently available. In a clinical situation, multiple positive and negative reinforcers are concurrently available under mutually exclusive, and non-exclusive contingencies. Although the current study represents a simplified choice environment, the present results expand our knowledge of the role of alternative reinforcers in opioid-taking behavior despite adverse consequences to include a negative reinforcer under both mutually exclusive and non-exclusive choice conditions. Elucidation of the neurobiological mechanisms by which alternative negative reinforcement may interact or compete with opioid-taking behavior is one future direction for this work [38].

## Supplementary information

Supplemental Materials
