# Supplementary material for: Effects of pharmacological and environmental manipulations on choice between fentanyl and shock avoidance/escape in male and female rats under mutually exclusive and non-exclusive choice conditions
Source: Neuropsychopharmacology. 2024 Aug 5;49(13):2011–21. doi: 10.1038/s41386-024-01939-7 (PMC11480371; doi:10.1038/s41386-024-01939-7)
Supplement: Supplementary file 1 — Supplemental Materials [file 41386_2024_1939_MOESM1_ESM.docx]

**Supplemental Materials for**

**Effects of pharmacological and environmental manipulations on choice between fentanyl and shock avoidance/escape in male and female rats under mutually exclusive and non-mutually exclusive choice conditions.**

Madison M. Marcus^1^, Samuel Marsh^1^, Michelle Arriaga^1^, Sidney S. Negus^1^, Matthew L. Banks^1,*^

^1^Department of Pharmacology and Toxicology, Virginia Commonwealth University School of Medicine, Richmond, VA, 23298 USA

**Methods:**

*Shock avoidance/escape training.* Rats were initially trained by hand using successive approximation to lever-press to escape electric foot shock during daily 30-min sessions consisting of 60 trials as previously described (Marcus & Banks, 2023). In each trial, a 3-s foot shock (0.4 mA) was presented along with the left lever and the associated white stimulus light above the lever. Responding was under a fixed ratio (FR) 1 schedule of reinforcement, such that a single response immediately terminated the shock, retracted the lever, and extinguished the stimulus light. The white house light was illuminated throughout all escape training sessions. Shock intensity was increased in 0.1 mA increments for each individual subject throughout training if the rat failed to exhibit a startle response to the shock stimulus. Acquisition criteria was defined as successful escape of ≥ 80% of the trials for three consecutive days. Once rats acquired escape responding, rats who would be tested in the mutually exclusive, discrete-trial, fentanyl-vs-shock avoidance/escape procedure were transitioned to an avoidance-training procedure that also consisted of 60 trials. Rats who would be tested in the non-exclusive, non-discrete trial fentanyl-vs-shock avoidance/escape procedure were transitioned to a non-discrete-trial avoidance training procedure. In the discrete-trial avoidance procedure, shock presentation was preceded by a 30-s avoidance period during which the left, avoidance/escape-associated lever was extended and associated white stimulus light was on. A single response (FR1) during the avoidance period cancelled the upcoming shock for that trial, retracted the left lever, and extinguished the avoidance/escape stimulus light. If the rat failed to emit a response during the avoidance period, a 3-s shock (0.7 mA) was presented. During shock presentation, the left lever remained extended, and the white avoidance/escape stimulus light remained illuminated, signaling the availability of an escape response. The number of escape and avoidance trails completed each day were recorded. In the non-discrete-trial avoidance training procedure, the left, avoidance/escape-associated lever was extended, and the discriminative stimulus light was turned on for the entire 30 min session. A 0.5-s foot shock occurred every 5-s (shock-shock interval 5 s) unless the rat lever-pressed on the shock avoidance/escape-associated lever (FR1) to re-set the shock countdown to 30s (response-shock interval 30 s). The number of responses on the shock avoidance/escape lever and the number of shocks received were recorded. Rats were trained on their respective avoidance procedure for a total of five consecutive days, rather than until reaching a specified acquisition criterion, based on the existing literature (Coyle et al., 1973; Kuribara & Tadokoro, 1984) and our previous study (Marcus & Banks, 2023) which indicate that only a small subset of rats tend to acquire avoidance responding.

*Fentanyl self-administration training.* Rats were trained to lever-press for an IV infusion of 3.2 µg/kg fentanyl on the right lever under an initial FR1 / 20-s time out schedule of reinforcement during daily 2-hr sessions as previously described (Townsend et al., 2021). Each session began with a non-contingent fentanyl infusion followed by a 60-s time out. The response period was signaled by extension of the right lever and illumination of the associated tricolor stimulus light above the lever. When response-requirement was met, the lever retracted, the stimulus light was extinguished, and an IV fentanyl infusion was administered. Once rats earned ≥ 10 fentanyl infusions during a 2-hr session, the FR requirement was increased to FR3. Training criteria was defined as ≥ 10 fentanyl infusions under an FR3 schedule of reinforcement for three days. There was no programmed limit to the total number of fentanyl infusions the rat could receive during the training session.

*Food-maintained responding training.* Rats were trained to lever press for a 5-s presentation of liquid food (32% chocolate-flavored Ensure™ diluted in water; Abbott Laboratories, Chicago, IL) on the left lever under an initial FR1 / 20-s time-out schedule of reinforcement during daily 2-hr sessions as previously described (Townsend et al., 2021). Each session began with a non-contingent food presentation followed by a 20-s time out. Liquid food availability was signaled by the illumination of the tricolor stimulus light above the right lever. After earning ≥ 30 food reinforcers during a 2-hr session, the FR requirement was increased to FR3. Acquisition criteria was defined as ≥ 30 food reinforcers under an FR3 schedule of reinforcement for three days.

Effects of acute diazepam on fentanyl-vs-shock avoidance/escape choice. An additional experiment determined acute diazepam effects (vehicle, 0.32 – 5.6 mg/kg) on fentanyl-vs- shock avoidance/escape choice. Benzodiazepines increase shock-punished opioid self-administration under single operant conditions (Panlilio et al., 2005), and we hypothesized that diazepam would also increase fentanyl choice when shock was instead scheduled as an alternative negative reinforcer. Diazepam doses and vehicle were counterbalanced between rats and administered intraperitoneally 10 minutes before the choice session. A one day “washout” period was incorporated between each vehicle or diazepam dose. Diazepam HCl solution (5mg/mL) was purchased from a commercial vendor (DASH Pharmaceuticals, Saddle River, NJ) and serially diluted in vehicle (40% propylene glycol, 10% ethanol, 50% water) for IP injection. Diazepam doses are expressed as the salt form listed above.

**Figures:**

Figure S1. The number of fentanyl, avoidance/escape, and omitted trials during the mutually exclusive fentanyl-vs-shock avoidance/escape choice procedure by males and females as a function of fentanyl dose. Abscissae: Unit fentanyl dose in micrograms per kilogram per infusion. Ordinates: number of (A) fentanyl trials (FR1), (B) shock avoidance/escape trials (0.7 mA, FR1), or (C) omitted trials. All points represent the mean ± SEM. N = 4-6F and 2-4M. ^#^Main effect of fentanyl dose, *Main effect of sex; Panel B: ^#^F(1.2, 6.0) = 18.0, p=0.0046; * F (1, 8) = 6.1, p=0.0389; Panel C: ^#^F (2, 10) = 19, p=0.0004; * F (1, 8) = 6.0, p=0.0398.

Figure S2. Effects of the presence or absence of concurrently available shock avoidance/escape contingency and associated discriminative stimuli on male and female fentanyl self-administration in mutually exclusive fentanyl-vs-shock avoidance/escape procedure. Abscissae: Presence (shock) or absence (no shock) of shock avoidance/escape contingency (0.5 mA, FR1). (A) Trials completed by males and females for 3.2 or (C) 10 μg/kg/infusion fentanyl. (B) Latency (s) for males and females to complete the first fentanyl only forced-trial for 3.2 or (D) 10 μg/kg/infusion fentanyl. All points represent the mean ± SEM, n = 4F and 4M. * significant effect of sex, ^#^ significant effect of shock condition. Panel A: ^#^F (1, 6) = 36.7, p = 0.0009; * F(1,6)= 11.6, p=0.0144. Panel B: ^#^F(1,6)=11.5, p=0.0146. Panel C: ^#^F(1,6)=5.4, p=0.0080. Panel D: ^#^F(1,6)=10.6, p=0.0173

Figure S3. Effects of acute diazepam (0.32 – 5.6 mg/kg, IP) pretreatment on fentanyl-vs-shock avoidance/escape choice in the mutually exclusive fentanyl-vs-shock avoidance/escape choice procedure. (A) trials completed for fentanyl (FR1), shock avoidance/escape (FR1), or omissions in a nine discrete-trial fentanyl-vs-shock avoidance/escape choice procedure. (B) Latency in s to complete the first fentanyl-only trial as a function of diazepam dose. Filled points denote significant (p < 0.05) difference compared to vehicle. All points represent mean ± SEM, n = 8 (4M/4F). Panel A shows 3.2 and 5.6 mg/kg diazepam significantly decreased shock avoidance/escape trials without altering fentanyl trials (diazepam dose: F (2.8, 27.9)=7.6, p=0.0009; reinforcer: F(1, 10)=17.6, p=0.002; dose × reinforcer: F(2.6, 22.5)=5.2, p=0.009). Omitted trials significantly increased with increasing diazepam doses (dose: F(2.8, 25.7)=17.9, p= 0.001). Panel B shows 0.32 mg/kg diazepam significantly decreased latency to complete the first fentanyl-only trial compared to vehicle, whereas 3.2 and 5.6 mg/kg diazepam significantly increased latency (diazepam dose: F(1.8, 12.8)=22.1, p=0.0001). The majority (7/8) of subjects reached the 1800-s limited hold after 5.6 mg/kg diazepam.


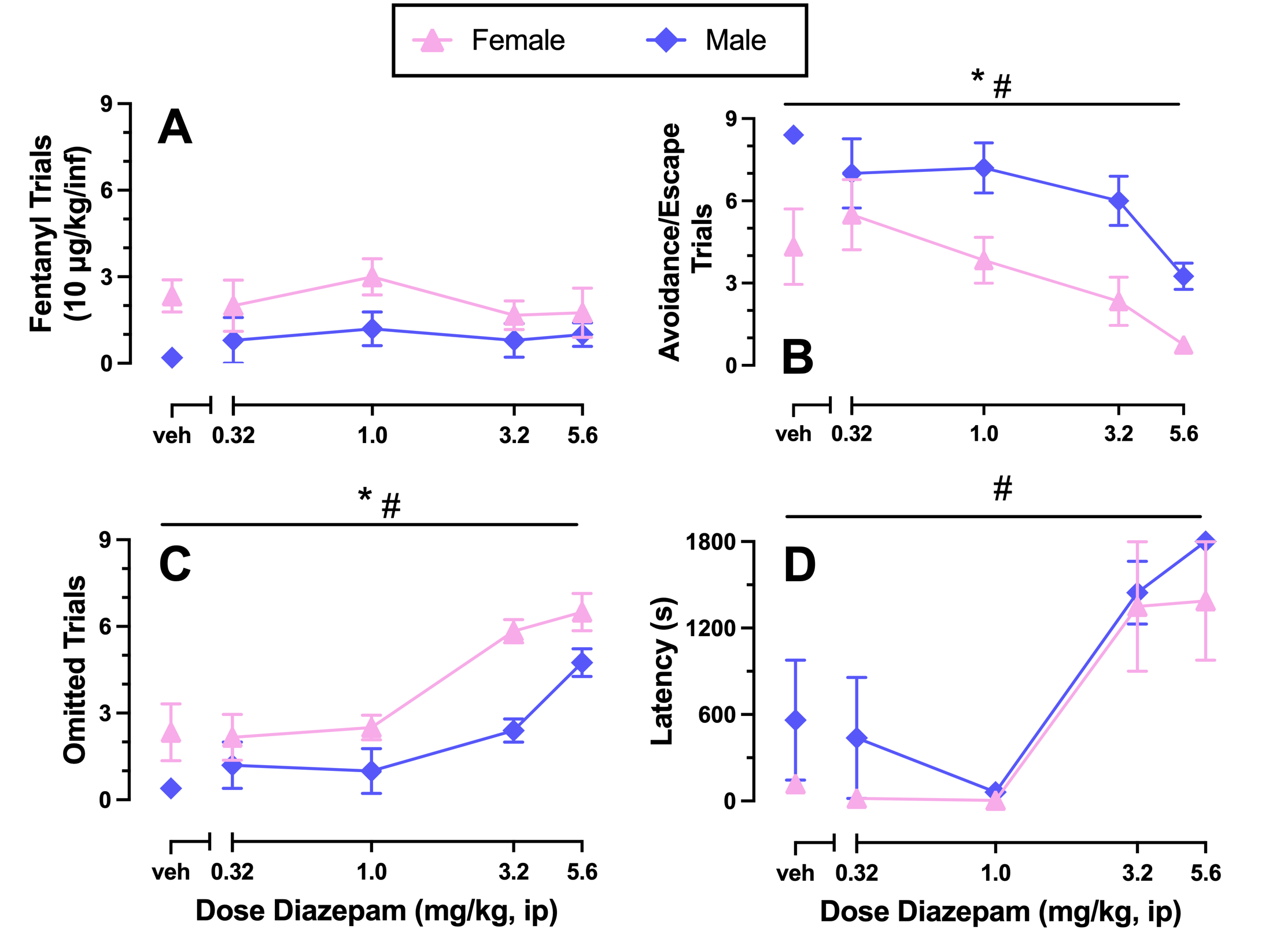


Figure S4. Effects of acute diazepam pretreatment on fentanyl-vs-shock avoidance/escape choice and start latency in males and females in the mutually exclusive choice procedure. Abscissae: Diazepam dose in milligrams per kilogram administered intraperitoneally. Ordinates: number of (A) fentanyl trials completed (10 μg/kg/infusion, FR1), (B) shock avoidance/escape trials completed (0.5 mA, FR1), (C) omitted trials, and (D) latency (s) to complete the first fentanyl-only forced trial. All points represent the mean ± SEM. N = 4-6F and 4-5M. * significant effect of sex, ^#^ significant effect of diazepam dose. Panel B: ^#^F(1.5,7.7)=8.2, p=0.02; *F(2,5)=11.5, p=0.02. Panel C: ^#^F(2,10)=21.8, p=0.0002; *F(1,5)=19.0, p=0.007. Panel D: ^#^F(2,6)=36.1, p=0.0004.

 Figure S5. Event log representing pattern of responding in the fentanyl-vs-shock avoidance/escape (30s R-S) non-exclusive choice procedure across the three fentanyl doses tested. Abscissae: Time (in seconds) of the choice procedure. Tick marks represent the event of a fentanyl infusion (orange), avoidance/escape response (blue), or shock (black) at a given time point. (A, B) At smaller doses of fentanyl (0.32 and 1.0 µg/kg/inf, respectively) the subject continues responding on both levers throughout entire 1hr procedure. (C) After earning several 3.2µg/kg/inf fentanyl infusions at approximately 3200s (53 min), the subject stops responding on either lever resulting in the delivery of many shocks. This aligns with experimenter observations of behavioral depression at large fentanyl doses.

Figure S6. Event log representing pattern of responding in the fentanyl-vs-shock avoidance/escape (100s R-S) non-exclusive choice procedure across the three fentanyl doses tested. Abscissae: Time (in seconds) of the choice procedure. Tick marks represent the event of a fentanyl infusion (orange), avoidance/escape response (blue), or shock (black) at a given time point. (A, B) At smaller doses of fentanyl (0.32 and 1.0 µg/kg/inf, respectively) the subject continues responding on both levers throughout entire 1hr procedure. (C) After earning several 3.2µg/kg/inf fentanyl infusions at approximately 2100s (35 min), the subject stops responding on either lever resulting in the delivery of hundreds of shocks. This aligns with experimenter observations of behavioral depression at large fentanyl doses.

Figure S7. The number of responses for fentanyl, shock avoidance/escape, and number of shocks received during the non-exclusive fentanyl-vs.-shock avoidance/escape choice procedure by males and females as a function of fentanyl dose, grouped by R-S interval. Abscissae: Unit fentanyl dose in micrograms per kilogram per infusion. Ordinates: number of (A) fentanyl trials (FR1), (B) shock avoidance/escape trials (FR1), or (C) number of shocks received. All points represent the mean ± SEM. N = 3F and 4M. Females took significantly more fentanyl infusions under 100s R-S conditions (F(1,3)=12.4, p=0.0388) and received significantly more shocks under 30s R-S conditions (F(1,3)=10.8, p=0.0463) compared to males.


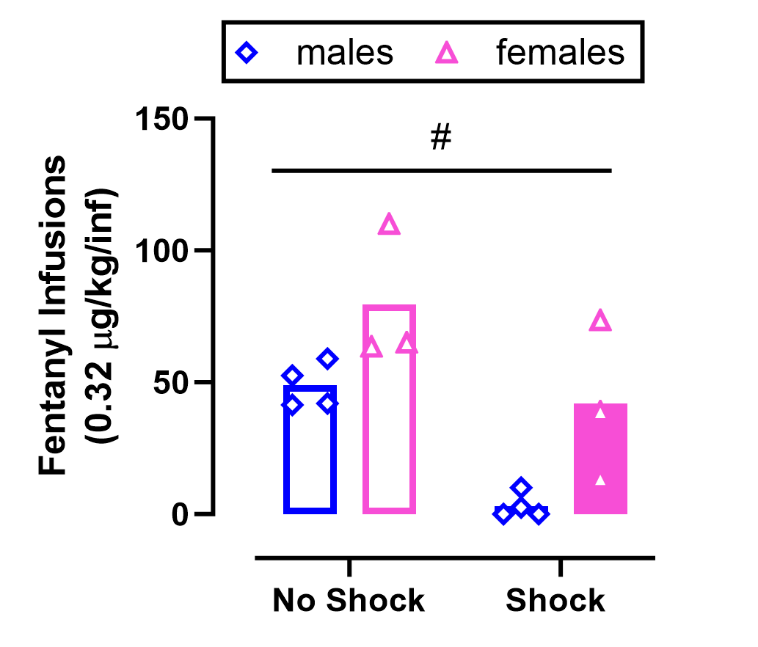


Figure S8. Effects of the presence or absence of concurrently available shock avoidance/escape contingency on male and female fentanyl self-administration (0.32 μg/kg/inf) in a non-exclusive fentanyl-vs-shock avoidance/escape choice procedure. ^#^Main effect of shock condition F(1, 5) = 146.2, p<0.0001


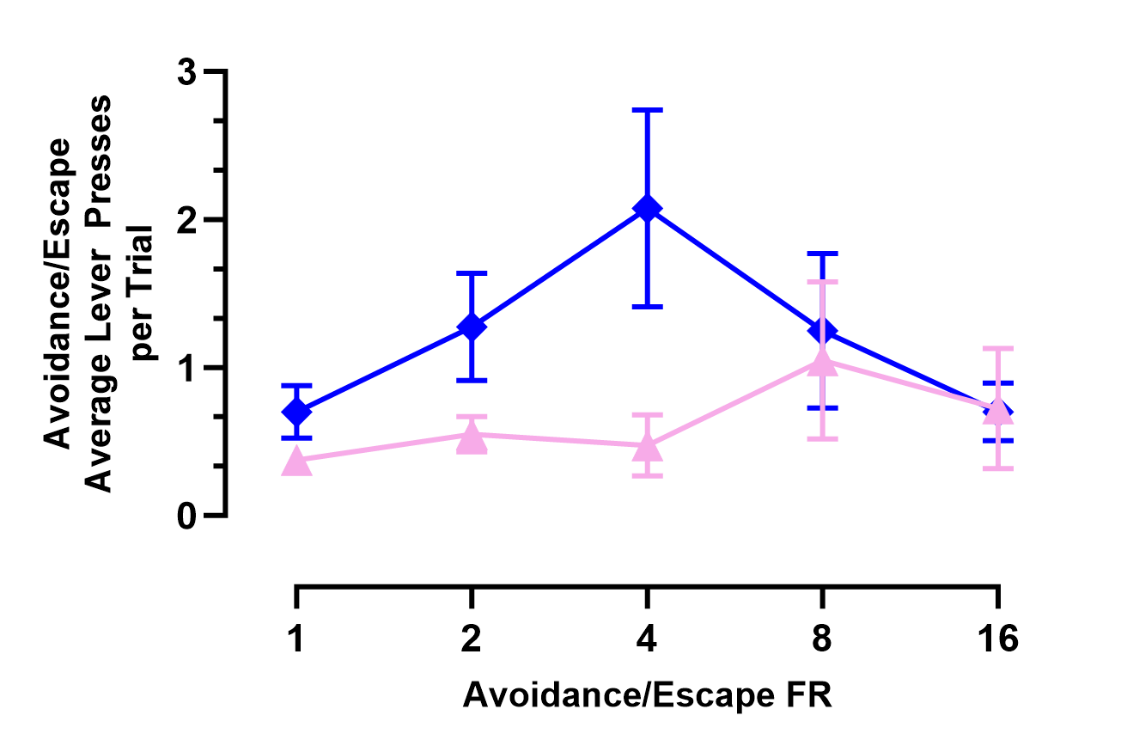


Figure S9. Effects of increasing the shock avoidance/escape response requirement on the average number of avoidance/escape lever presses per trial in males and females in the mutually exclusive choice procedure. All points represent the mean ± SEM, n = 4F and 4M. There were no significant comparisons.


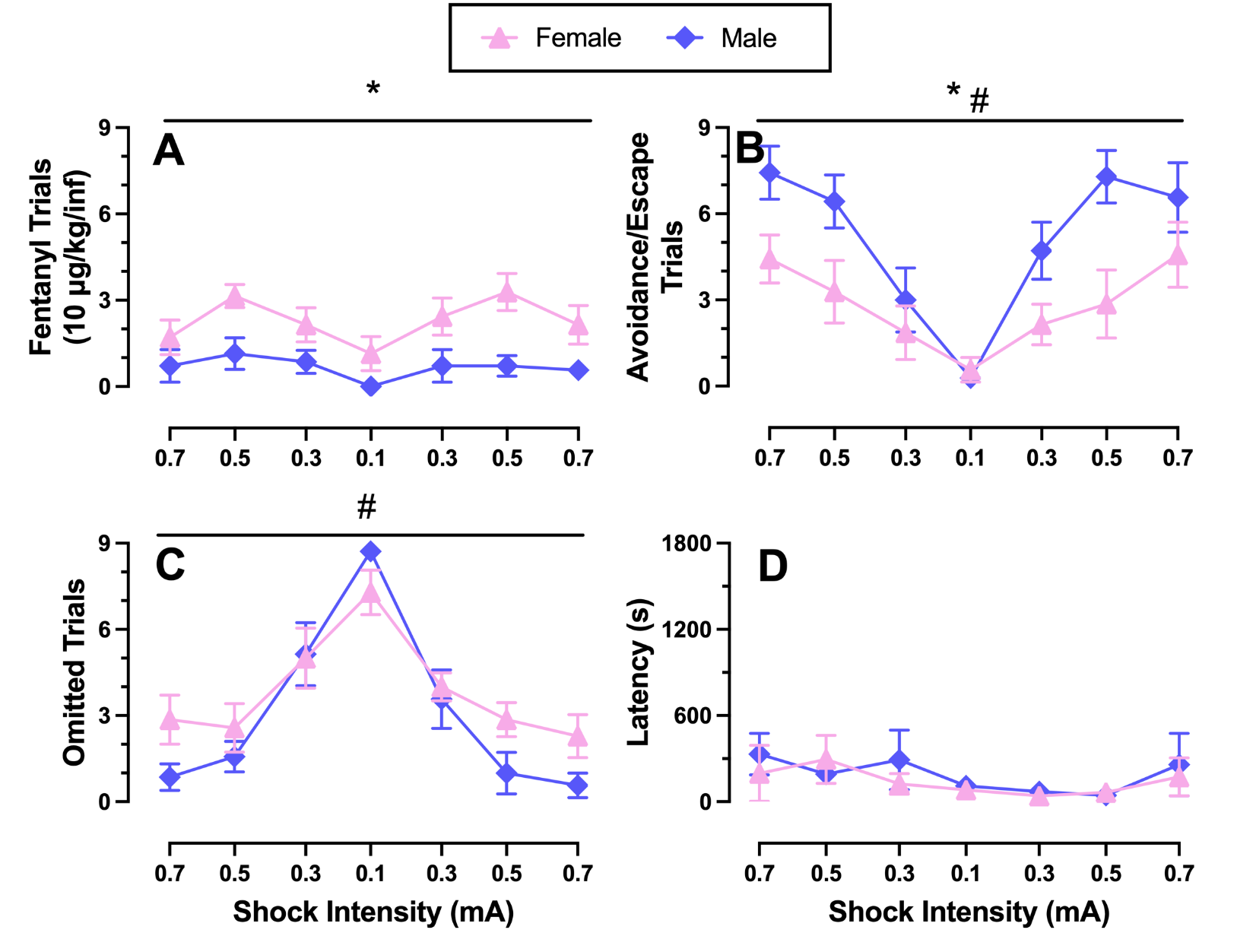


Figure S10. Effects of shock intensity on fentanyl-vs-shock avoidance/escape choice and start latency in males and females in the mutually exclusive choice procedure. Abscissae: shock magnitude in milliamps. Ordinates: number of (A) fentanyl trials (10 μg/kg/infusion, FR1), (B) shock avoidance/escape trials (0.7, 0.5, 0.3, 0.1 mA, FR1), (C) omitted trials; (D) latency (s) to complete the first fentanyl-only forced-trial. All points represent the mean ± SEM, n = 7F and 7M. * significant effect of sex, ^#^ significant effect of shock intensity. Panel A: * F(1, 6)= 14.2, p=0.01; Panel B: ^#^F(3.3, 19.7)=9.5, p=0.0003, *F(1,6)=17.4, p=0.006; Panel C: ^#^F(2.8, 16.5)=18.4, p<0.0001.


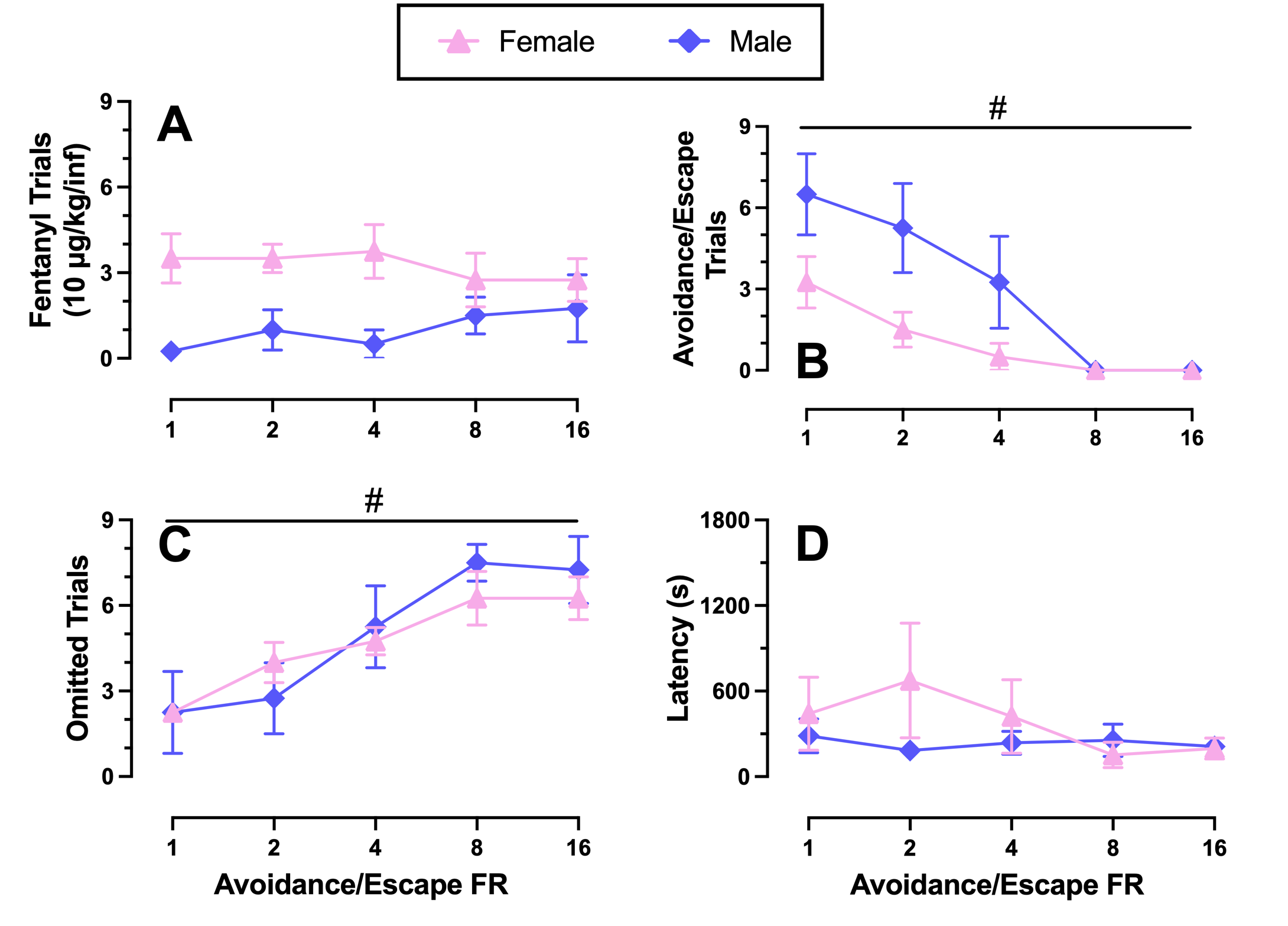


Figure S11. Effects of increasing the shock avoidance/escape response requirement on fentanyl-vs-shock avoidance/escape choice and start latency in males and females in the mutually exclusive choice procedure. Abscissae: negative reinforcer fixed ratio. Ordinates: number of (A) fentanyl trials completed (10 μg/kg/infusion, FR1), (B) shock avoidance/escape trials completed (0.5 mA, FR1), (C) omitted trials, and (D) latency (s) to complete the first fentanyl-only forced-trial. All points represent the mean ± SEM, n = 4F and 4M. ^#^ denotes significant effect of avoidance/escape response requirement. Panel B: ^#^F(4,12)=13.8, p=0.0002; Panel C: ^#^F(2.5, 7.6)=13.7, p=0.002.

Figure S12. Effects of increasing the response-shock (R-S) interval on non-exclusive fentanyl-vs-shock avoidance/escape choice in males and females. Abscissae: R-S interval in seconds. Ordinates: (A) fentanyl infusions (0.32 μg/kg/infusion, FR1), (B) shock avoidance/escape responses (FR1), (C) percent fentanyl choice and (D) shocks received. Points represent individual subjects, bars represent group mean, n = 4M/3F. # Main effect of R-S interval, *Main effect of sex, ^$^R-S interval × sex interaction. Panel A: ^#^F (1.6, 8.0) = 8.8, p=0.0120; Panel B: ^#^F(1.4, 7.0) = 34.8, p=0.0004; Panel C: ^#^F (2.1, 10.7) = 27.7, p<0.0001; Panel D: ^#^ F (1.4, 6.8) = 27.0, p=0.0009, * F(1, 5) = 8.6, p=0.0327, ^$^ F(3, 15) = 8.4, p=0.0017. No significant differences were detected when post-hoc tests that corrected for multiple comparisons were used.


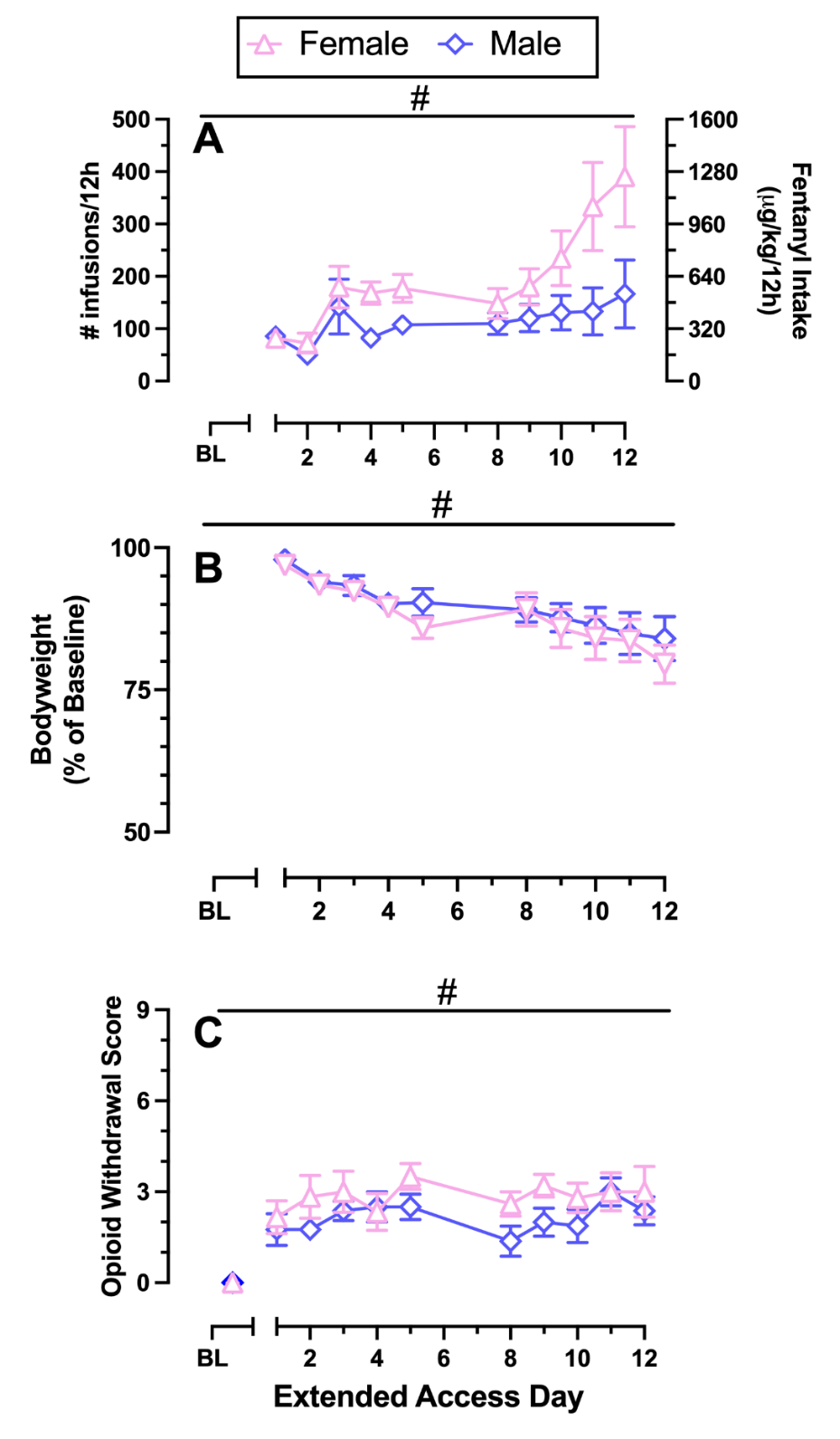


Figure S13. Fentanyl self-administration by males and females under extended-access conditions in the mutually exclusive choice procedure. Abscissae: Extended Access Day. Ordinates: (A) total number of 3.2 μg/kg fentanyl infusions earned over 12-h extended-access session and corresponding fentanyl intake in micrograms per kilogram, (B) changes in bodyweight, relative to baseline, and (C) opioid somatic withdrawal signs. All points represent the mean ± SEM, n = 5-6F and 8M. ^#^denotes significant effect of extended access day. Panel A: ^#^F(2.1, 17.1)=8.1, p=0.003. Panel B: ^#^F(1.5, 11.7)=20.9, p=0.0003. Panel C: ^#^F(3.8, 30.7)=7.4, p=0.0003.


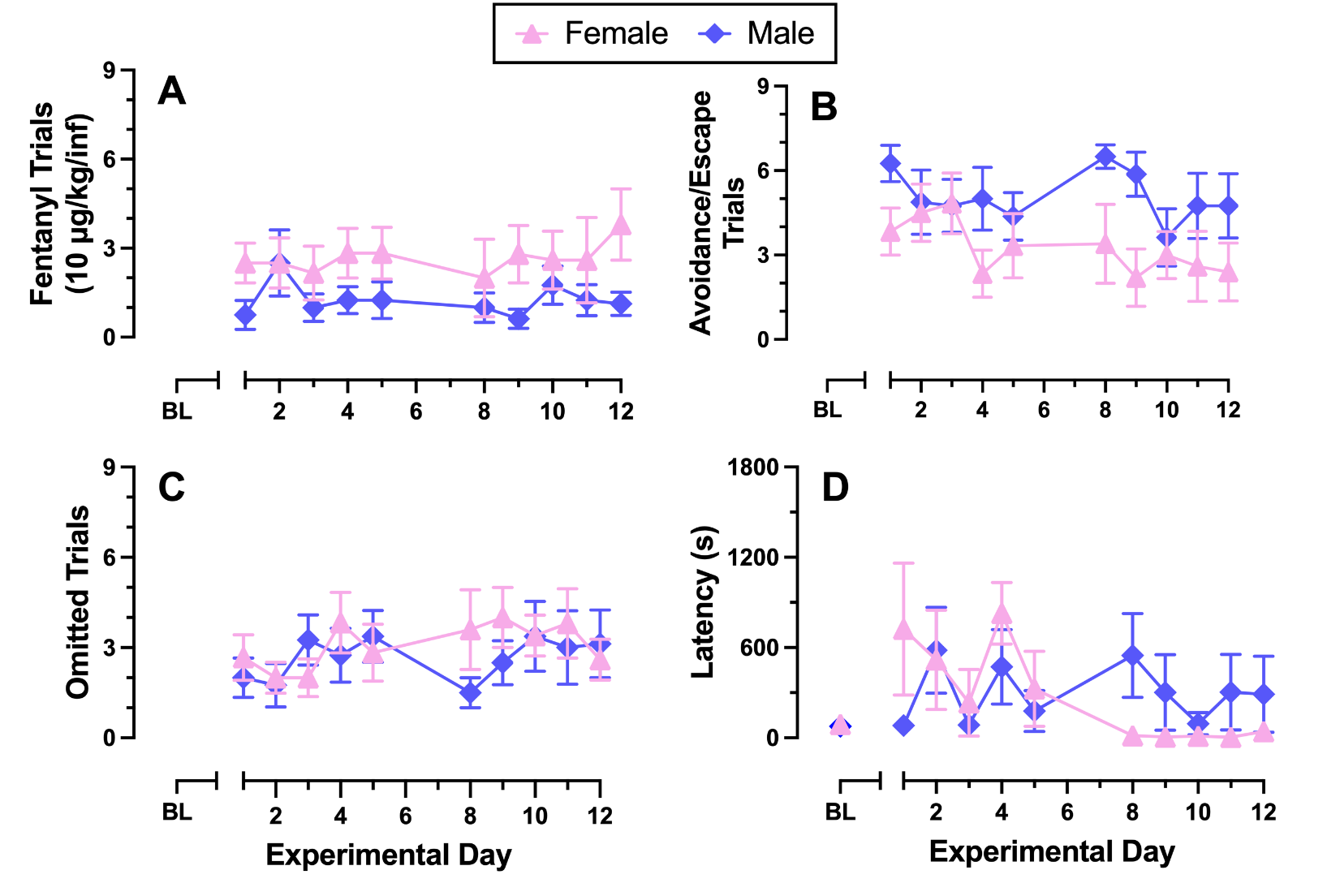


Figure S14. Effects of extended fentanyl access on fentanyl-vs-shock avoidance/escape choice and start latency in males and females. Abscissae: extended access day. Ordinates: number of (A) fentanyl trials completed (10 μg/kg/infusion, FR1), (B) shock avoidance/escape trials completed (0.5 mA, FR1), (C) omitted trials, and (D) latency (s) to complete the first fentanyl-only forced trial. All points represent the mean ± SEM, n = 5-6F and 8M. There were no significant differences detected.

Figure S15. Fentanyl self-administration by males and females under extended-access conditions in the non-exclusive choice procedure. Abscissae: Extended Access Day. Ordinates: (A) total number of 3.2 μg/kg fentanyl infusions earned over 12-h extended-access session and corresponding fentanyl intake in micrograms per kilogram, (B) changes in bodyweight, relative to baseline, and (C) opioid somatic withdrawal signs. All points represent the mean ± SEM, n = 3F/4M. ^#^Main effect of EA day; *Main effect of sex; ^$^Sex × Day interaction. Panel A: ^#^F (9, 45) = 11.57, p<0.0001; Panel B: ^#^F (1.5, 7.3) = 63.4, p<0.0001; *F (1, 5) = 13.8, p=0.0137; ^$^F (10, 50) = 5.6, p<0.0001. No significant multiple comparisons. Panel C: ^#^F (3.3, 16.5) = 5.7, p=0.0062.

Figure S16. Effects of extended fentanyl access on fentanyl-vs-shock avoidance/escape choice in a non-exclusive choice procedure in male subjects (n=4). Abscissae: response-shock interval. (A) percent fentanyl choice, (B) shocks received, (C) fentanyl infusions, (D) shock avoidance/escape responses. ^#^Main effect of R-S interval. Points represent individual subject data, whereas bars represent group mean. Panel A: ^#^F(1.5, 4.5) = 18.8, p=0.0078; Panel B: ^#^F(1.2, 3.6) = 16.2, p=0.0185; Panel D: ^#^F(1.0, 3.1) = 15.9, p=0.0271.

 Figure S17. Effects of extended fentanyl access on fentanyl-vs-shock avoidance/escape choice in a non-exclusive choice procedure in female subjects (n=3). Abscissae: response-shock interval. (A) percent fentanyl choice, (B) shocks received, (C) fentanyl infusions, (D) shock avoidance/escape responses. Points represent individual subject data, whereas bars represent group mean. No significant comparisons were detected.

**Supplemental Material References:**

Coyle, J. T., Wender, P., & Lipsky, A. (1973). Avoidance conditioning in different strains of rats: Neurochemical correlates. *Psychopharmacologia*, *31*, 25–34.

Kuribara, H., & Tadokoro, S. (1984). Conditioned lever-press avoidance response in mice: acquisition processes and effects of diazepam. *Psychopharmacology*, *82*, 36–40.

Marcus, M. M., & Banks, M. L. (2023). Effects of environmental and pharmacological manipulations on cocaine-vs-negative reinforcer choice in male and female rats. *Psychopharmacology*, *240*, 1677–1689. https://doi.org/10.1007/s00213-023-06404-9

Panlilio, L. V., Thorndike, E. B., & Schindler, C. W. (2005). Lorazepam reinstates punishment-suppressed remifentanil self-administration in rats. *Psychopharmacology*, *179*(2), 374–382. https://doi.org/10.1007/s00213-004-2040-2

Townsend, Schwienteck, K. L., Robinson, H. L., Lawson, S. T., & Banks, M. L. (2021). A drug-vs-food “choice” self-administration procedure in rats to investigate pharmacological and environmental mechanisms of substance use disorders. *J Neurosci Meth*, *354*, 109110. https://doi.org/10.1016/j.jneumeth.2021.109110
